# Matrix metalloproteinase-10 protects against acute kidney injury by augmenting epidermal growth factor receptor signaling

**DOI:** 10.1038/s41419-020-03301-3

**Published:** 2021-01-12

**Authors:** Chengxiao Hu, Yangyang Zuo, Qian Ren, Xiaoli Sun, Shan Zhou, Jinlin Liao, Xue Hong, Jinhua Miao, Lili Zhou, Youhua Liu

**Affiliations:** 1 State Key Laboratory of Organ Failure Research, National Clinical Research Center of Kidney Disease, Division of Nephrology, Nanfang Hospital, Southern Medical University, Guangzhou, China; 2 Department of Pathology, University of Pittsburgh School of Medicine, Pittsburgh, PA USA

**Keywords:** Apoptosis, Cell growth

## Abstract

Matrix metalloproteinase-10 (MMP-10) is a zinc-dependent endopeptidase involved in regulating a wide range of biologic processes, such as apoptosis, cell proliferation, and tissue remodeling. However, the role of MMP-10 in the pathogenesis of acute kidney injury (AKI) is unknown. In this study, we show that MMP-10 was upregulated in the kidneys and predominantly localized in the tubular epithelium in various models of AKI induced by ischemia/reperfusion (IR) or cisplatin. Overexpression of exogenous MMP-10 ameliorated AKI, manifested by decreased serum creatinine, blood urea nitrogen, tubular injury and apoptosis, and increased tubular regeneration. Conversely, knockdown of endogenous MMP-10 expression aggravated kidney injury. Interestingly, alleviation of AKI by MMP-10 in vivo was associated with the activation of epidermal growth factor receptor (EGFR) and its downstream AKT and extracellular signal-regulated kinase-1 and 2 (ERK1/2) signaling. Blockade of EGFR signaling by erlotinib abolished the MMP-10-mediated renal protection after AKI. In vitro, MMP-10 potentiated EGFR activation and protected kidney tubular cells against apoptosis induced by hypoxia/reoxygenation or cisplatin. MMP-10 was colocalized with heparin-binding EGF-like growth factor (HB-EGF) in vivo and activated it by a process of proteolytical cleavage in vitro. These studies identify HB-EGF as a previously unrecognized substrate of MMP-10. Our findings also underscore that MMP-10 can protect against AKI by augmenting EGFR signaling, leading to promotion of tubular cell survival and proliferation after injury.

## Introduction

Acute kidney injury (AKI) is characterized by an abrupt loss of kidney function. AKI is highly prevalent, particularly among hospitalized patients^1,2^, and it is associated with high morbidity and mortality^2,3^. Despite intensive studies over the past several decades, the underlying mechanism of AKI remains poorly understood^4,5^. There are no specific and effective remedies for treating patients with AKI in the clinic, and current therapy is largely supportive^6^. In this context, delineation of the mechanisms of AKI is paramount for understanding its pathophysiology and developing therapeutic strategies.

Matrix metalloproteinase-10 (MMP-10) belongs to a family of the zinc-dependent endopeptidases involved in regulating a wide variety of biologic processes, such as cell survival, proliferation, and migration, as well as remodeling of the extracellular matrix (ECM)^7–10^. In addition to proteolytically breaking down ECM proteins, MMPs are known to be capable of cleaving a number of non-ECM substrates, thereby eliciting much broader biological actions^8,11,12^. Several MMPs such as MMP-2, MMP-7, and MMP-9 have been implicated in regulating kidney injury and repair after AKI^13–16^. MMP-10 is involved in the development of glomerular disease and emerges as a potential therapeutic target for slowing the progression of diabetic nephropathy^9,10^. MMP-10 expression is also increased in renal cell carcinoma (RCC) and its levels are associated with poor prognosis and decreased 5-years survival of RCC patients^17^. However, the role of MMP-10 in the pathogenesis of AKI is completely unknown.

In this study, we investigated MMP-10 expression and its function in both ischemic and toxic models of AKI. Our studies illustrate that MMP-10 is induced in kidney tubular epithelium in various models of AKI and functionally renal protective. We demonstrate that MMP-10 protects against AKI by promoting tubular cell survival and proliferation via proteolytically cleaving heparin-binding epidermal growth factor-like growth factor (HB-EGF). These studies identify HB-EGF as a novel substrate of MMP-10 and underscore a critical role of EGF receptor (EGFR) activation in promoting tubular repair and regeneration after AKI.

## Materials and methods

### Mouse models of AKI

Male C57BL/6 mice, at age of 8 weeks old, were purchased from the Southern Medical University Animal Center (Guangzhou, China) and housed at the animal research facility of Nanfang Hospital, Southern Medical University. Mice were acclimatized to the laboratory condition and provided free access to food and water. For ischemic AKI model, mice were subjected to bilateraand l renal IRI by an established protocol as described previously^18,19^. The ischemia time was 30 min in the set of experiments involving MMP-10 overexpression, whereas the duration of ischemia was 27 min in the sets of experiments involving MMP-10-shRNA. For toxic AKI, mice were subjected to a single intraperitoneal injection of cisplatin (Sigma, St. Louis, MO) at a dose of 20 mg/kg as described elsewhere^20^. To induce rhabdomyolysis, the animals were intramuscularly injected in each thigh caudal muscle with 50% glycerol at 7.5 ml/kg or saline as controls^21^. All animal experiments were approved by the Animal Ethic Committee at the Nanfang Hospital, Southern Medical University.

### In vivo expression or knockdown of MMP-10 and pharmacologic inhibition of EGFR in mice

For studying the effects of MMP-10, four sets of experiments were performed. The detailed experimental designs were presented in the corresponding figures. In vivo expression or knockdown of MMP-10 in mice was carried out by a hydrodynamic-based gene delivery approach, as described previously^18^. Mouse MMP-10 shRNA plasmid (pLVX-MMP-10-shR) was constructed by ligating MMP-10 siRNA sequences (5'CCAGCTAACTTCCACCTTT3') into the shRNA expression plasmid (pLVX-shRNA). For pharmacologic inhibition of EGFR signaling, mice were administered with erlotinib (80 mg/kg by gavages) or vehicle daily beginning 1 day prior to surgery, as previously reported^22^.

### Determination of serum BUN and creatinine

Serum creatinine and BUN levels were determined by an automatic chemistry analyzer. The levels of serum creatinine and BUN were expressed as mg/dl.

### Histology and immunohistochemical staining

Paraffin-embedded mouse kidney sections were prepared by a routine procedure. The sections were stained with periodic acid-Schiff staining reagents by standard protocol. Immunohistochemical staining was performed using routine protocol. Antibodies used were described in the Supplementary Table S1. The staining was assessed semi-quantitatively in a blinded fashion.

### TUNEL assays

TUNEL staining for apoptotic cells was performed on the paraffin-embedded kidney sections using a standard commercial kit. Results were expressed as the average number of TUNEL-positive cells per high-powered field (HPF).

### Western blot analysis

Protein expression was analyzed by western blot analysis as described previously^23^. The primary antibodies used were described in the Supplementary Table S1.

### qPCR

Total RNA was prepared using TRIzol RNA isolation system. The first strand of complementary DNA was synthesized using GoScript™ reverse transcription system (Promega) according to the manufacturer’s instruction. RT-PCR amplification was performed using a GoTaq Green Master Mix kit (Promega). Quantitative, real-time PCR (qPCR) was performed using the SYBR Select Master Mix (Invitrogen). The sequences of the primer pairs were described in the Supplementary Table S2.

### Cell culture and treatment

Human proximal tubular epithelial cells (HKC-8) were cultivated according to the procedures described previously^13^. HKC-8 cells were treated with cisplatin at 25 µg/ml to induce cell apoptosis. For some experiments, HKC-8 cells were pretreated with human recombinant MMP-10 (R&D Systems) at 100 ng/ml for 1 h, followed by incubation with cisplatin. Some cells were also harvested and analyzed with PE-conjugated Annexin V (AV)-labeled apoptotic cells detection kit. To knockdown HB-EGF, HKC-8 cells were transiently transfected with control- or HB-EGF-specific siRNA using Lipofectamine 2000 reagent according to the protocol specified by the manufacturer (Invitrogen, Grand Island, NY). The target sequence used for knockdown of HB-EGF in this study was as follows: 5′-CCCUUUGGAGAAUGCAGUUTT-3′.

### Hypoxia/reoxygenation injury

In vitro ischemia model of HKC-8 cells was established by hypoxia/reoxygenation protocol^18^. After pretreatment with rhMMP-10 at 100 ng/ml or 2 µM erlotinib for 1 h, cells were incubated in glucose-free medium in a tri-gas incubator (94% N2, 5% CO_2_, and 1.0% O_2_) at 37 °C for 24 h. Subsequently, cells were incubated in complete medium under normal (5% CO_2_, and 95% air) conditions for 4 h for reoxygenation, and then harvested for various analyses.

### In vitro assay for cleavage and release of soluble HB-EGF

To establish an in vitro assay for identifying whether MMP-10 can cleave and release soluble HB-EGF, we transfected HKC-8 cells with HB-EGF-GFP fusion protein expression vector (pHB-EGF-GFP). The fusion protein in HKC-8 cell lysates was then immunoprecipitated and incubated with rhMMP-10 for 2 h. After MMP-10 incubation, the supernatant and the beads were subsequently analyzed by western blot analyses. In another set of experiment, HKC-8 cells were transfected with pHB-EGF-GFP for 48 h. After incubation with rhMMP-10 for 2 h, these cells were harvested for western blot analyses to detect the phosphorylation of EGFR and ERK1/2.

### Statistical analyses

All data examined were expressed as mean ± SEM. Statistical analysis of the data was carried out using SPSS 20.0 (SPSS Inc, Chicago, IL). Comparison between groups was made using one-way ANOVA followed by Student-Newman-Kuels test or Dunnett’s T3 procedure. *P* < 0.05 was considered significant.

## Results

### MMP-10 is induced in kidneys after AKI

**Fig. 1:**
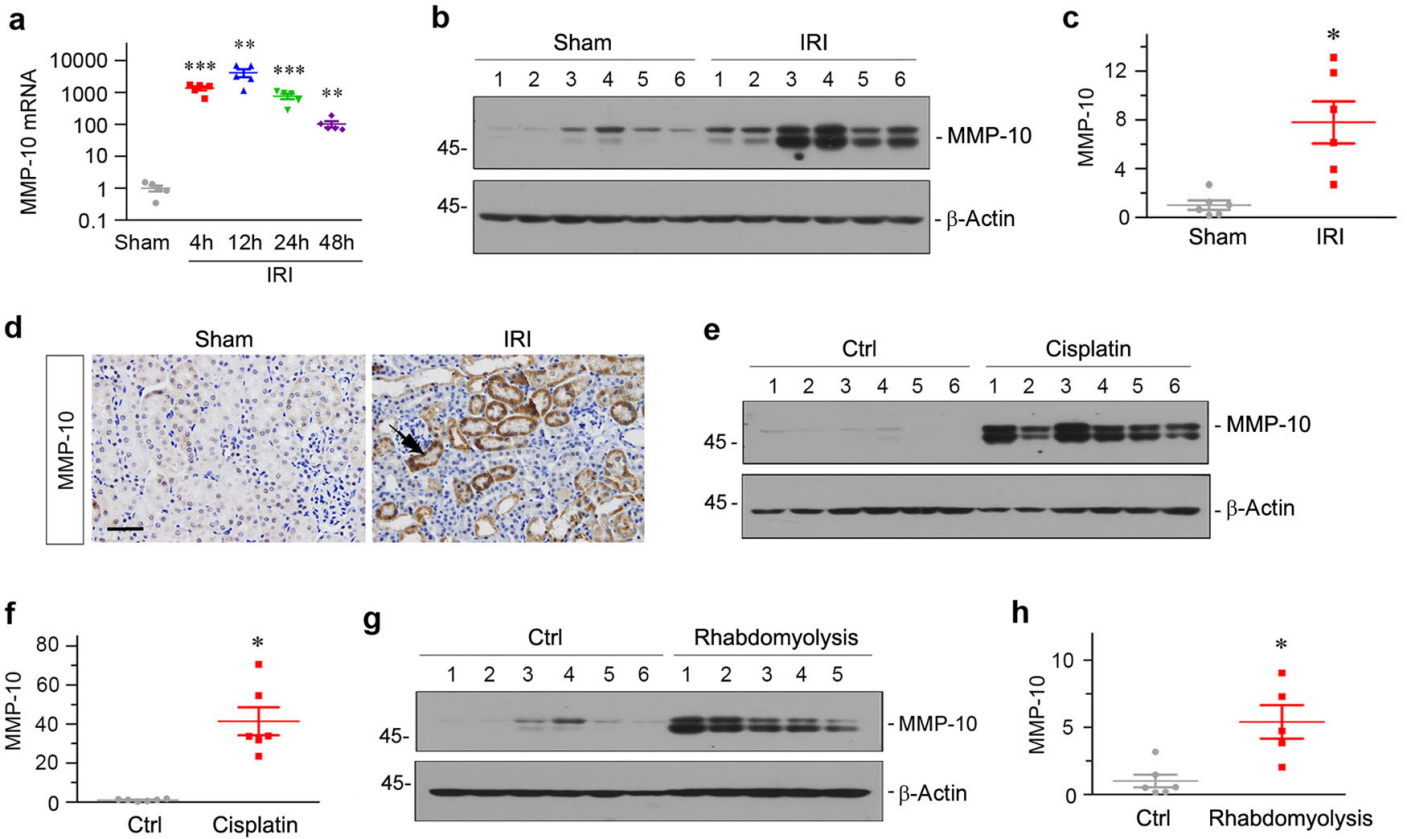
Matrix metalloproteinase-10 (MMP-10) is induced in various models of AKI. **a** Quantitative, real-time PCR (qPCR) results show induction of renal MMP-10 mRNA at different time points after IRI as indicated. Relative abundances of renal MMP-10 mRNA were assessed by qPCR, and fold induction over the sham controls was reported. **P < 0.01, ***P < 0.001 versus sham controls (*n* = 5). **b**, **c** Western blot analyses show renal expression of MMP-10 protein at 24 hours after IRI. Western blot (**b**) and quantitative data (**c**) are presented. Numbers (1–6) indicate each individual animal in a given group. *P < 0.05 vs sham controls. **d** Representative micrographs show MMP-10 protein localization in sham and ischemic kidneys at 24 h after IRI. Kidney sections were immunohistochemically stained with specific antibody against MMP-10 (*n* = 6). Arrow indicates positive staining. Scale bar, 50 µm. **e**, **f** Western blot (**e**) and quantitative data (**f**) of r**e**nal MMP-10 protein in the kidneys after cisplatin injury were presented. Kidney samples were collected at 3 days after cisplatin injection. Numbers (1–6) indicate each individual animal in a given group. *P < 0.05 versus controls. (**g**, **h**) Western blot (**g**) and quantitative data (**h**) show renal MMP-10 expression at 30 h after injection of glycerol. Numbers (1–6) indicate each individual animal in a given group. *P < 0.05 versus controls.

We first examined renal expression of MMP-10 in various models of AKI. As shown in Fig. 1a, renal expression of MMP-10 mRNA was upregulated as early as 4 h and sustained at least to 48 h after ischemia-reperfusion injury (IRI), as revealed by quantitative, real-time PCR (qPCR) analyses. Accordingly, western blotting showed an increased expression of MMP-10 protein at 1 day after IRI (Fig. 1b, c). Similar results were obtained when MMP-10 protein was assessed by immunohistochemical staining. As shown in Fig. 1d, MMP-10 was predominantly expressed in renal tubular epithelium after IRI.

We then assessed the expression of MMP-10 in other models of AKI. As shown in Fig. 1e, f, MMP-10 protein expression was also upregulated in the kidneys after injection of cisplatin. Consistently, renal expression of MMP-10 was significantly induced in mouse rhabdomyolysis-associated AKI induced by glycerol (Fig. 1g, h). Together, these results indicate that MMP-10 induction is a common pathologic finding in various models of AKI.

### Expression of exogenous MMP-10 protects against AKI after IRI

**Fig. 2:**
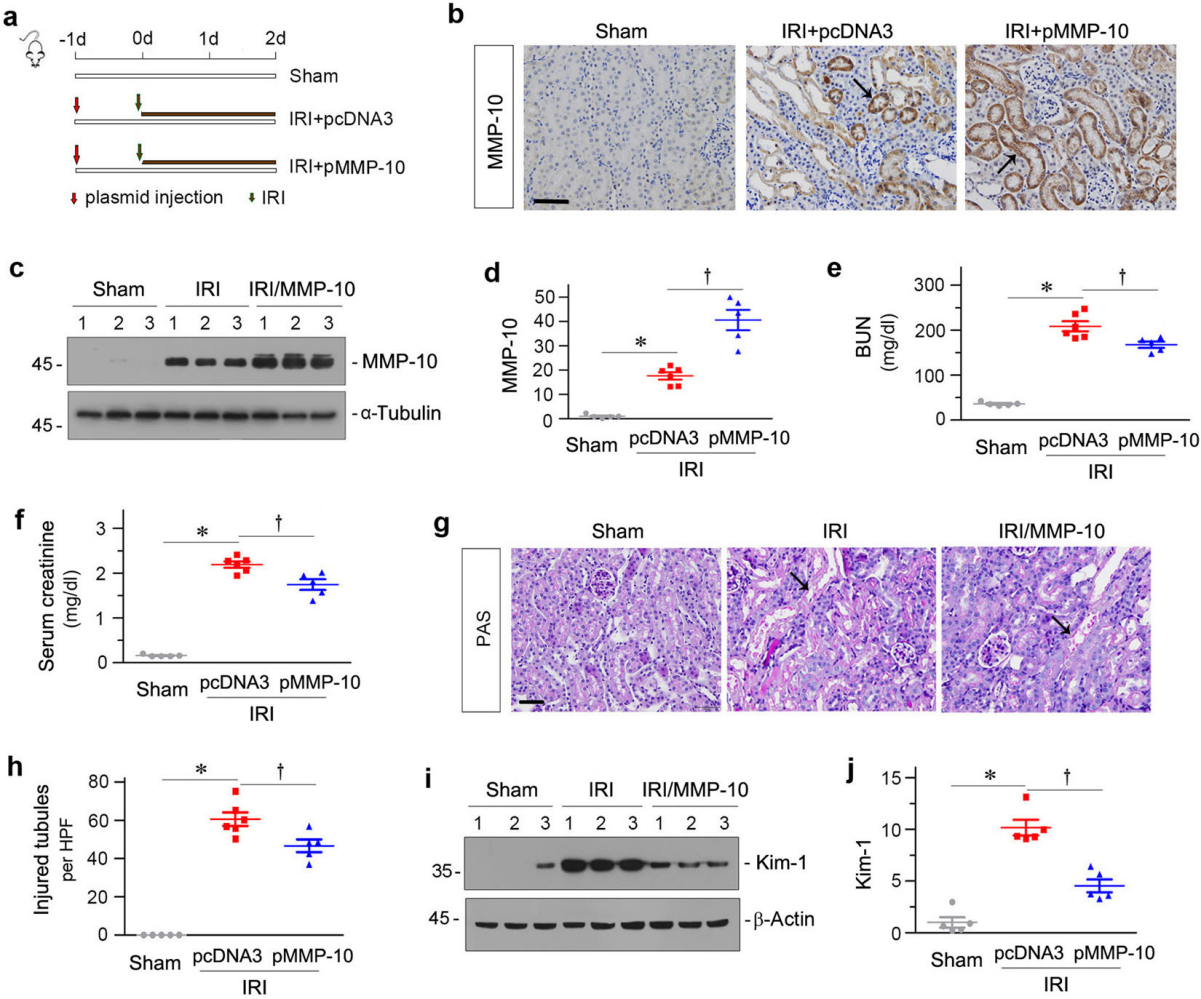
Expression of exogenous MMP-10 protects mice against AKI after IRI. **a** Experimental design. Red arrows indicate the injection of pcDNA3 or pMMP-10 plasmids. Green arrows indicate the timing of renal IRI surgery. **b** Representative micrographs show renal MMP-10 protein expression in different groups, as indicated. Arrow indicates positive staining. Scale bar, 50 µm. **c**, **d** Representative Western blot (**c**) and quantitative data (**d**) show the abundances of renal MMP-10 expression protein in different groups as indicated. Numbers (1–3) indicate each individual animal in a given group. *P < 0.05 versus sham controls (*n* = 5–6); ^†^*P* < 0.05 versus IRI injected with pcDNA3 (*n* = 5–6). **e**, **f** Graphic presentations show blood urea nitrogen (BUN) (**e**) and serum creatinin**e** (**f**) levels in different groups as indicated. *P < 0.05 versus sham controls (*n* = 5–6); ^†^*P* < 0.05 versus IRI injected with pcDNA3 (*n* = 5–6). **g** Representative micrographs show kidney morphology after IRI in different groups of mice as indicated. Kidney sections were subjected to PAS staining. Kidney sections were stained with PAS reagents. Arrows indicate injured tubules. Scale bar, 50 µm. **h** Quantitative analyses of injured tubules in three groups as indicated. At least 10 randomly selected fields in the cortex-medulla junctional area were evaluated and results were averaged for each kidney. *P < 0.05 versus sham controls; ^†^*P* < 0.05 versus IRI injected with pcDNA3 (*n* = 5–6). **i**, **j** Representative western blot (**i**) and quantitative data (**j**) show renal Kim-1 expression. Numbers (1–3) indicate each individual animal in a given group. *P < 0.05 versus sham controls (*n* = 5–6); ^†^*P* < 0.05 versus IRI injected with pcDNA3 (*n* = 5–6).

To investigate the function of MMP-10 in AKI, we sought to overexpress exogenous MMP-10 by delivering a Flag-tagged MMP-10 expression vector (pMMP-10) via a hydrodynamic-based gene transfer approach^24,25^. Figure 2a shows the detailed experimental protocols. As shown in Fig. 2b–d, immunohistochemical staining and western blotting revealed an increased expression of MMP-10 protein in renal tubular epithelium after injection of pMMP-10 vector.

We next assessed the effect of exogenous MMP-10 on kidney function after IRI. As shown in Fig. 2e, f, expression of exogenous MMP-10 reduced blood urea nitrogen (BUN) and serum creatinine levels, suggesting a reno-protective role of MMP-10 in AKI. We further examined kidney histopathology by periodic acid-Schiff (PAS) staining. As shown in Fig. 2g, h, expression of MMP-10 ameliorated renal tubular injury characterized by loss of brush border, tubular cell death and detachment, denuded tubular basement membrane, cast formation in tubular lumen after IRI. Similar results were obtained when Kim-1, a tubular injury marker, was assessed by western blot analyses (Fig. 2i, j).

### MMP-10 inhibits tubular cell apoptosis and promotes their proliferation and regeneration

**Fig. 3:**
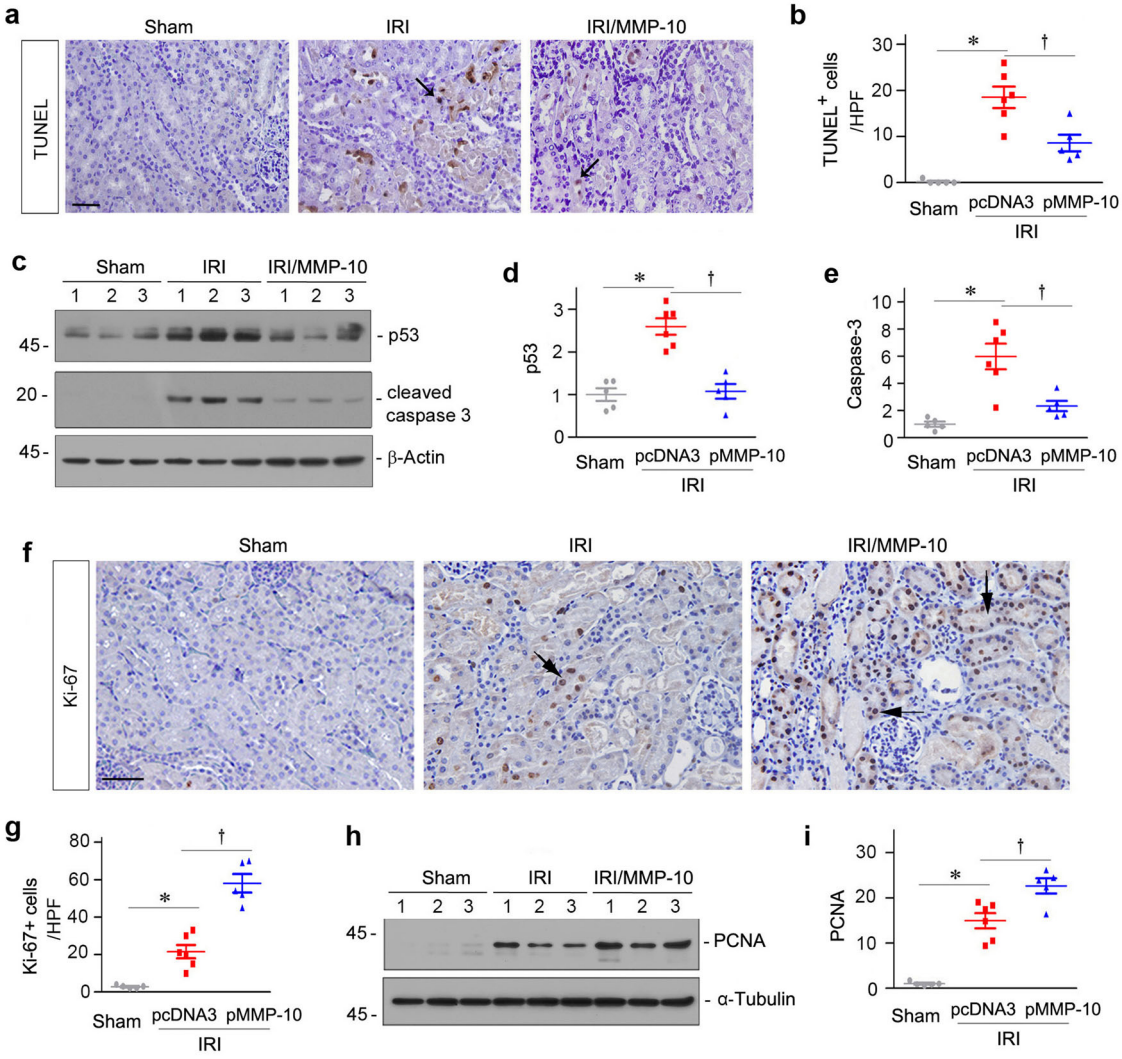
Exogenous MMP-10 inhibits tubular cell apoptosis and promotes tubular repair after IRI. **a** Representative micrographs show TUNEL^+^ cells in different groups as indicated. Arrows indicate positive staining. Scale bar, 50 µm. **b** Graphic presentation shows the quantification of TUNEL^+^ cells in different groups as indicated. At least 10 randomly selected high-power fields (HPF) were evaluated and results were averaged for each kidney. *P < 0.05 versus sham controls; ^†^*P* < 0.05 versus IRI injected with pcDNA3 (*n* = 5–6). **c** Representative western blots show renal expression of p53 and cleaved caspase-3 in different groups as indicated. Numbers (1, 2, and 3) indicate each individual animal in a given group. **d**, **e** Graphic presentations show the relative abundance of renal p53 (**d**) and cleaved caspase-3 (**e**) in different groups. *P < 0.05 versus sham controls (*n* = 5–6); ^†^*P* < 0.05 versus IRI injected with pcDNA3 (*n* = 5–6). **f** Representative micrographs show Ki-67 expression in different groups as indicated. Kidney sections were immunostained with a specific antibody against Ki-67. Arrows indicate positive staining. Scale bar, 50 µm. **g** Quantitative determination of Ki-67^+^ cells is presented. At least 10 randomly selected fields were assessed and results averaged for each kidney. *P < 0.05 versus sham controls (*n* = 5–6); ^†^*P* < 0.05 versus IRI injected with pcDNA3 (*n* = 5–6). **h** Representative western blots show renal expression of PCNA protein in different groups. Numbers (1, 2, and 3) indicate each individual animal in a given group. **i** Graphic presentations show the relative abundances of renal PCNA protein in different groups. *P < 0.05 versus sham controls (*n* = 5–6); ^†^*P* < 0.05 versus IRI injected with pcDNA3 (*n* = 5–6).

We further assessed the effects of MMP-10 on kidney tubular cell apoptosis by terminal deoxynucleotidyl transferase-mediated dUTP nick end-labeling (TUNEL) staining. As shown in Fig. 3a, apoptotic cells were evident in the kidneys after IRI, predominantly in renal tubular epithelium. However, expression of exogenous MMP-10 reduced tubular cell apoptosis (Fig. 3a, b). We next examined renal expression of apoptosis-regulatory protein p53 by western blot analyses. As shown in Fig. 3c, d, renal expression of p53 proteins was increased in IRI mice, which was inhibited by MMP-10 expression. Similar results were observed when cleaved caspase-3 was assessed (Fig. 3c, e).

We next examined the effect of MMP-10 on tubular cell proliferation and regeneration after AKI, the major cellular events in kidney recovery^26^. As shown in Fig. 3f, g, immunostaining for Ki-67, a known marker of cell proliferation, showed that ectopic expression of MMP-10 promoted tubular cell proliferation after IRI. Furthermore, western blot analyses showed an induction of the proliferating cell nuclear antigen (PCNA), another cell proliferation marker, in the ischemic kidneys after expression of exogenous MMP-10 (Fig. 3h, i).

### Knockdown of endogenous MMP-10 aggravates AKI after IRI

**Fig. 4:**
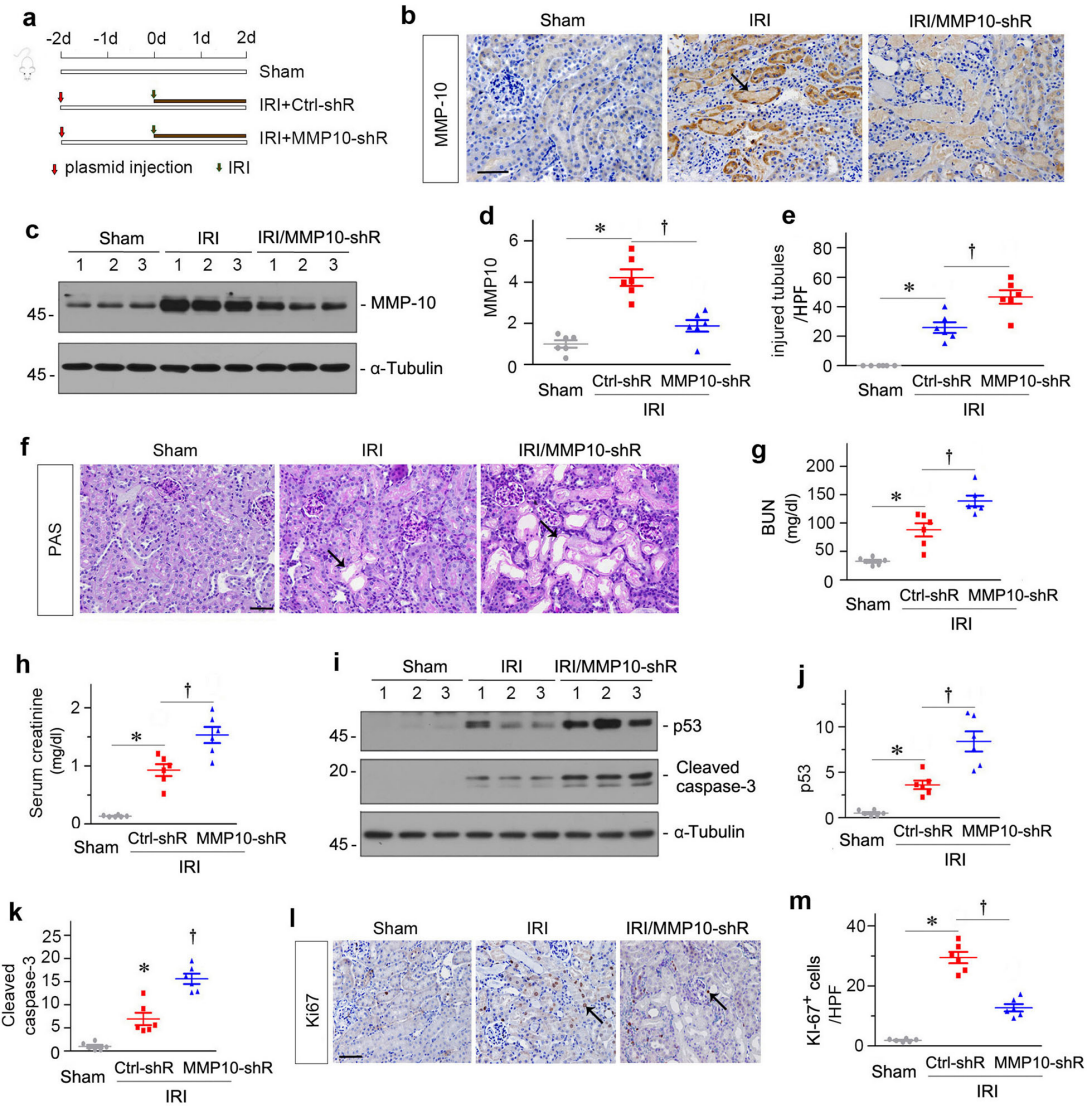
Knockdown of MMP-10 aggravates kidney injury after IRI. **a** Experimental design. Red arrows indicate the injection of Ctrl-shR and MMP-10-shR plasmids. Green arrows indicate the timing of IRI surgery. **b** Representative micrographs show renal MMP-10 expression in different groups as indicated. Arrow indicates positive staining of MMP-10 expression protein. Scale bar, 50 µm. **c** Representative western blots show renal MMP-10 protein expression in different groups as indicated. **d** Graphic presentation shows the relative MMP-10 protein levels in different groups. *P < 0.05 versus sham controls (*n* = 6); ^†^*P* < 0.05 versus IRI injected with Ctrl-shR (*n* = 6). **e** Representative micrographs show kidney morphology in different groups of mice as indicated. Kidney sections were subjected to PAS staining. Arrows indicate injured tubules. Scale bar, 50 µm. **f** Quantitative analyses of injured tubules in three groups. At least 10 randomly selected high-power fields (HPF) were evaluated and results were averaged for each kidney. *P < 0.05 versus sham controls (*n* = 6); ^†^*P* < 0.05 versus IRI injected with Ctrl-shR (*n* = 6). **g**, **h** Graphic presentations show blood urea nitrogen (BUN) (**g**) and serum creatinine (**h**) levels in different groups as indicated. *P < 0.05 versus sham controls (*n* = 6); ^†^*P* < 0.05 versus IRI injected with Ctrl-shR (*n* = 6). **i** Representative western blots show renal expression of p53 and cleaved caspase-3 in different groups as indicated. Numbers (1, 2, and 3) indicate each individual animal in a given group. **j**, **k** Graphic presentations show the relative abundance of renal p53 (**j**) and cleaved caspase-3 (**k**) proteins in different groups. *P < 0.05 versus sham controls (*n* = 6); ^†^*P* < 0.05 versus IRI injected with Ctrl-shR (*n* = 6). **l** Representative micrographs show Ki-67 protein expression in different groups as indicated. Kidney sections were immunostained with specific antibody against Ki-67. Arrows indicate positive staining. Scale bar, 50 µm. **m** Quantitative determination of Ki-67^+^ cells is presented. At least 10 randomly selected high-power fields (HPF) were evaluated and results were averaged for each kidney. *P < 0.05 versus sham controls (*n* = 6); ^†^*P* < 0.05 versus IRI injected with Ctrl-shR (*n* = 6).

To confirm the protective role of MMP-10 in AKI, we utilized an opposite strategy by knocking down endogenous MMP-10 expression in vivo. To this end, mice were intravenously injected with a short hairpin RNA (shRNA) vector (pLVX-shMMP-10) to silence MMP-10 expression in vivo via a hydrodynamic-based gene delivery approach. Two days after injection, mice were subjected to IRI and analyzed at 48 h after surgery (Fig. 4a). As shown in Fig. 4b–d, both immunohistochemical staining and Western blot analyses showed that renal expression of MMP-10 was downregulated in IRI mice after intravenous injection of MMP-10 shRNA plasmid.

We next assessed the morphologic and functional impact of MMP-10 depletion on the kidneys after IRI. As shown in Fig. 4e, f, knockdown of MMP-10 significantly exacerbated the pathohistological changes induced by IRI. Consistent with these results, levels of BUN and serum creatinine were further increased after depletion of MMP-10 in IRI mice (Fig. 4g, h). We also examined renal expression of apoptosis-related proteins by western blotting. As shown in Fig. 4i–k, renal p53 and cleaved caspase-3 expression were induced in IRI mice and knockdown of MMP-10 further promoted their expression. Furthermore, knockdown of MMP-10 also reduced Ki-67^+^ tubular cells in the kidneys after IRI (Fig. 4l, m). Therefore, depletion of endogenous MMP-10 aggravates kidney injury by promoting apoptosis and inhibiting tubular regeneration after IRI.

### Exogenous MMP-10 ameliorates nephrotoxic AKI induced by cisplatin

To further study the role of MMP-10 in AKI, we adopted a nephrotoxic AKI model induced by cisplatin. The experimental design was shown in Supplemental Fig. S1a. Mice were intraperitoneally injected with cisplatin 24 h after injection of pFlag-MMP-10 plasmid by a hydrodynamic-based gene delivery. As shown in Supplemental Fig. S1b, at 3 days after injection of cisplatin, kidney developed significant injury characterized by tubular dilation, hyaline casts, and tubular cell death and detachment. However, expression of exogenous MMP-10 protected kidneys against these injuries. Quantification of tubular lesions also confirmed the protective effect of MMP-10 on cisplatin-induced tubular injury (Supplemental Fig. S1c). Consistently, expression of exogenous MMP-10 also decreased BUN and serum creatinine levels in mice after cisplatin injection (Supplemental Fig. S1d and e).

We then assessed tubular cell injury and apoptosis by examining renal expression of Kim-1, p53 and cleaved caspase-3. As shown in Supplemental Fig. S1f–i, cisplatin significantly induced the expression of these proteins. However, exogenous MMP-10 blunted the induction of these injury-associated proteins. Furthermore, exogenous MMP-10 also promoted renal expression of PCNA in cisplatin-treated mice (Supplemental Fig. S1f and j). Similarly, immunohistochemical staining for Ki-67 also showed that exogenous MMP-10 promoted tubular cell proliferation after cisplatin injury (Supplemental Fig. S1k and l). These results suggest that MMP-10 promotes cell proliferation and regeneration in nephrotoxic AKI as well.

### MMP-10 activates EGFR signaling

**Fig. 5:**
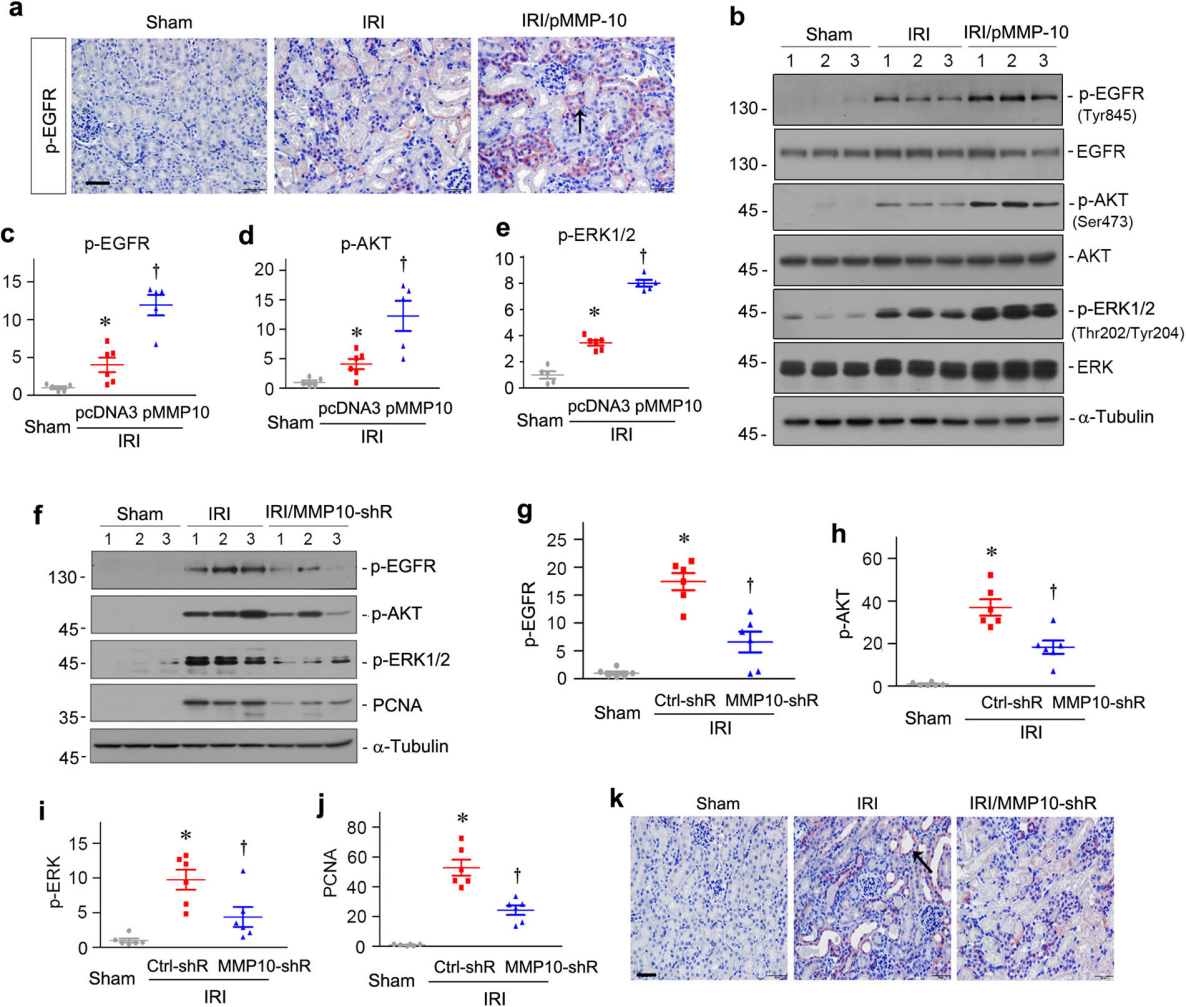
MMP-10 activates epidermal growth factor receptor (EGFR) signaling. **a** Micrographs show that exogenous MMP-10 promoted renal expression of phosphorylated EGFR after IRI. Representative image from five animals (*n* = 5). Arrow indicates positive staining of phosphorylated EGFR (Tyr845). Scale bar, 50 µm. **b** Representative western blots show renal expression of p-EGFR (Tyr845), total EGFR, p-AKT (Ser473), total AKT, p-ERK1/2 (Thr202/Tyr204), and total ERK1/2 in different groups as indicated. Numbers (1, 2, and 3) indicate each individual animal in a given group. **c**–**e** Graphic presentations show the quantitative data of p-EGFR (**c**), p-AKT (**d**) and p-ERK1/2 (**e**) in different groups as indicated. *P < 0.05 versus sham controls (*n* = 5–6); ^†^*P* < 0.05 versus IRI injected with pcDNA3 (*n* = 5–6). **f** Knockdown of MMP-10 inhibited EGFR signaling after IRI. Representative western blots show renal expression of p-EGFR, p-AKT, p-ERK1/2, and PCNA in different groups as indicated. Numbers (1, 2, and 3) indicate each individual animal in a given group. **g**–**j** Graphic presentations show the quantitative data of p-EGFR (Tyr845) (**g**), p-AKT (Ser473) (**h**), p-ERK1/2 (Thr202/Tyr204) (**i**), and PCNA (**j**) in different groups as indicated. *P < 0.05 versus sham controls (*n* = 6); ^†^*P* < 0.05 versus IRI injected with Ctrl-shR (*n* = 6). **k** Representative micrographs show renal expression of p-EGFR in different groups as indicated (*n* = 6). Arrow indicates positive staining. Scale bar, 50 µm.

To delineate the mechanism underlying MMP-10 protection against AKI, we examined EGFR signaling, as earlier studies have implicated it in mediating renal protection against AKI^27,28^. As shown in Fig. 5a, exogenous MMP-10 promoted EGFR phosphorylation at tyrosine 845 (p-EGFR) in the survived renal tubules after IRI, as illustrated by immunohistochemical staining. Similarly, western blot analyses also revealed an increased renal expression of p-EGFR (Tyr845) in IRI mice, which was further augmented after exogenous MMP-10 expression (Fig. 5b, c). We further examined the downstream effectors of EGFR signal cascade including AKT kinase and extracellular signal-regulated kinase-1 and -2 (ERK1/2). As shown in Fig. 5b, d, e, IRI induced renal AKT (Ser473) and ERK1/2 (Thr202/Tyr204) phosphorylation and activation, which was further enhanced by MMP-10 expression. Of note, either IRI or MMP-10 did not affect the levels of total EGFR, AKT, and ERK1/2 in the kidney (Fig. 5b).

We next examined the effect of MMP-10 depletion on EGFR signaling in the kidneys after IRI. As shown in Fig. 5f–i, knockdown of MMP-10 largely blocked the upregulation of p-EGFR and inhibited the activation of its downstream effectors AKT and ERK1/2 induced by IRI. Furthermore, depletion of MMP-10 also resulted in a decreased PCNA expression in the kidneys after IRI (Fig. 5f, j). Consistently, immunostaining also revealed that knockdown of MMP-10 abolished p-EGFR expression in renal tubules after IRI (Fig. 5k), suggesting a close correlation between MMP-10 expression and EGFR activation.

### Inhibition of EGFR abolishes renal protection of MMP-10 after AKI

**Fig. 6:**
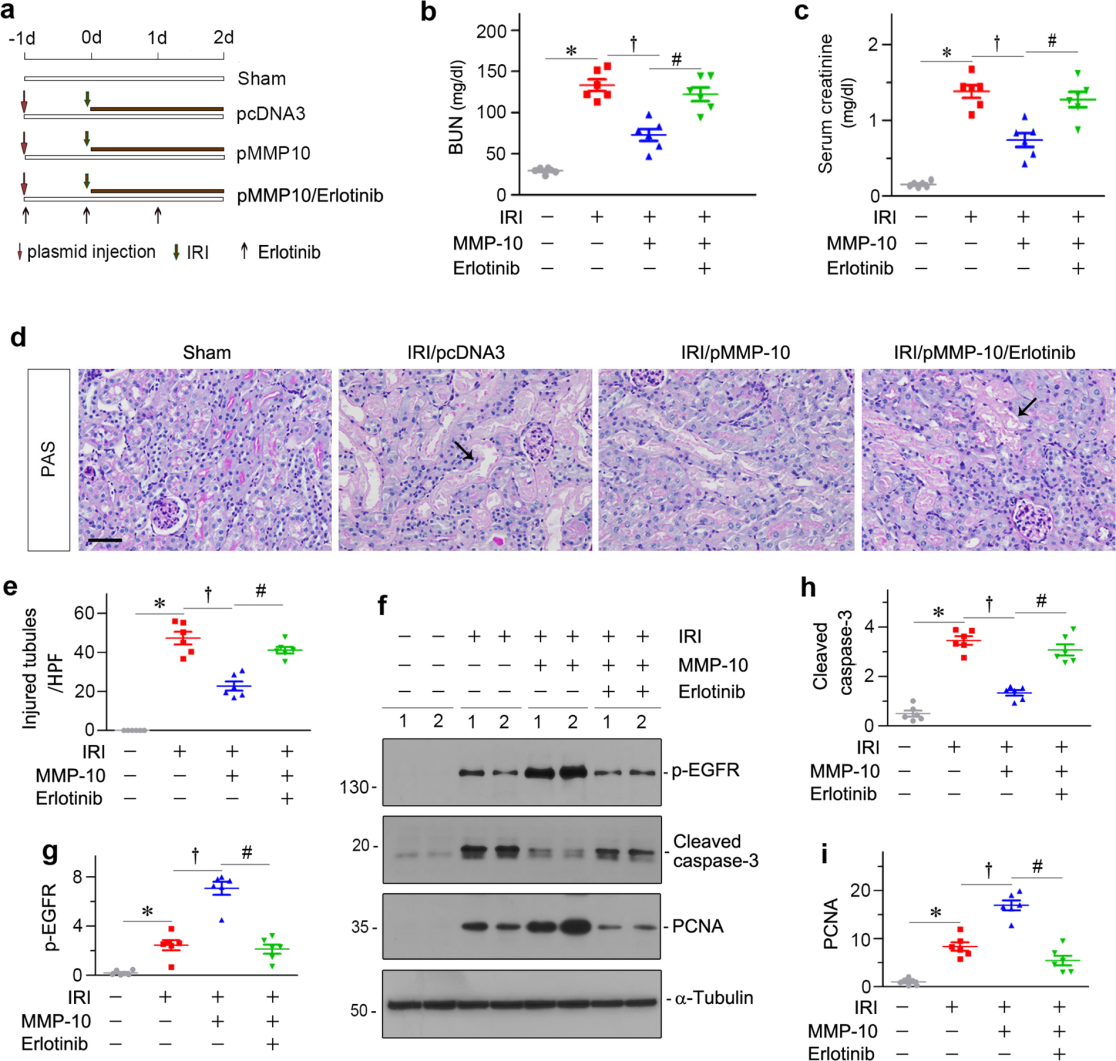
Pharmacologic inhibition of EGFR signaling abolishes the protective role of exogenous MMP-10 after IRI. **a** Experimental design. Red arrows indicate the injection of plasmids. Green arrows indicate the timing of IRI surgery. Black arrows indicate the oral gavage of erlotinib. **b**, **c** Graphic presentations show blood urea nitrogen (BUN) (**b**) and serum creatinine (**c**) levels in different groups as indicated. *P < 0.05 versus sham controls (*n* = 6); ^†^*P* < 0.05 versus IRI injected with pcDNA3 (*n* = 6). ^#^*P* < 0.05 versus IRI injected with pMMP-10 (*n* = 6). **d** Representative micrographs show kidney morphology at 2 days after IRI in different groups as indicated. Arrows indicate injured tubules. Scale bar, 50 µm. **e** Quantitative determination of injured tubules in different groups is shown. Kidney sections were subjected to PAS staining. At least 10 randomly selected high-power fields were evaluated and results averaged for each kidney. *P < 0.05 versus sham controls (*n* = 6); ^†^*P* < 0.05 versus IRI injected with pcDNA3 (*n* = 6); ^#^*P* < 0.05 versus IRI injected with pMMP-10 (*n* = 6). **f** Representative western blots show renal expression of p-EGFR, cleaved caspase-3 and PCNA in different groups as indicated. Numbers (1 and 2) indicate each individual animal in a given group. **g**–**i** Graphic presentations show the relative abundances of renal p-EGFR (**g**), cleaved caspase-3 (**h**), and PCNA (**i**) proteins in different groups as indicated. *P < 0.05 versus sham controls (*n* = 6); ^†^*P* < 0.05 versus IRI injected with pcDNA3 (*n* = 6); ^#^*P* < 0.05 versus IRI injected with pMMP-10 (*n* = 6).

To confirm the role of EGFR activation in mediating MMP-10-triggered renal protection against AKI, we treated mice with erlotinib, a specific FDA-approved EGFR tyrosine kinase inhibitor^29^. The detail experimental design was shown in Fig. 6a. As shown in Fig. 6b, c, the levels of BUN and serum creatinine were decreased after injection of pMMP-10 plasmid in IRI mice; however, inhibition of EGFR signaling by erlotinib largely abolished the protective effects of MMP-10 on renal function. Consistently, erlotinib also eradicated the protective effects of MMP-10 on kidney morphology, as shown by kidney histology and quantitative determination of tubular injury (Fig. 6d, e).

We next investigated the effect of erlotinib on EGFR signaling, cell apoptosis and tubular regeneration in IRI mice. As shown in Fig. 6f, g, exogenous MMP-10 induced renal EGFR activation in IRI mice, which was abolished by erlotinib treatment. Similarly, western blot analyses revealed that erlotinib restored renal expression of cleaved caspse-3 and inhibited PCNA expression induced by MMP-10 in IRI mice (Fig. 6f, h, i). These data suggest that EGFR activation mediates renal protective actions of MMP-10 in vivo.

### MMP-10 prevents tubular cell apoptosis by augmenting EGFR signaling in vitro

**Fig. 7:**
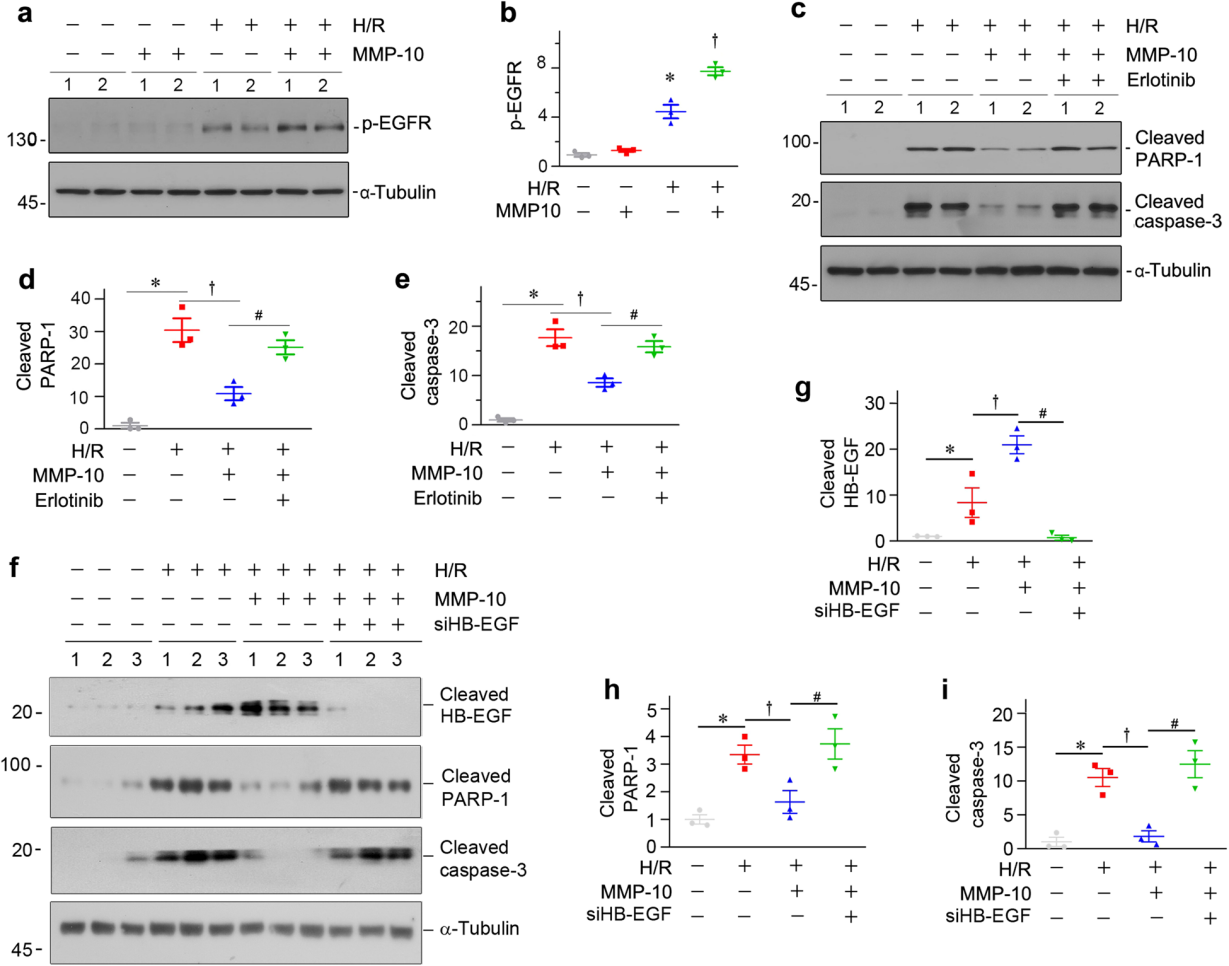
MMP-10 promotes tubular cell survival through augmenting EGFR signaling in vitro. **a** Representative western blots show protein expression of p-EGFR after various treatments in HKC-8 cells. **b** Graphic presentations show the relative abundances of p-EGFR in different groups as indicated. *P < 0.05 versus control cells; ^†^*P* < 0.05 versus H/R alone (*n* = 3). **c** Representative Western blots show protein expression of cleaved PARP-1 and cleaved caspase-3 after various treatments in HKC-8 cells. **d**, **e** Graphic presentations show the relative abundances of cleaved PARP-1 (**d**) and cleaved caspase-3 (**e**) proteins in different groups as indicated. *P < 0.05 versus control cells; ^†^*P* < 0.05 versus H/R alone; ^#^*P* < 0.05 versus H/R with MMP-10 (*n* = 3). **f**–**i** Knockdown of HB-EGF by siRNA abolishes MMP-10-mediated protection of tubular epithelial cells against apoptosis induced by hypoxia/reoxygenation (H/R). Western blots (**f**) and quantitative data show protein expression of cleaved HB-EGF (**g**), cleaved PARP-1 (**h**), and cleaved caspase-3 (**i**) after various treatments as indicated in HKC-8 cells.

We next investigated the role of MMP-10 in tubular cell apoptosis in vitro. To imitate IRI in vitro, we treated human proximal tubular epithelial cells (HKC-8) using the hypoxia-reoxygenation (H/R) protocol, as described previously^18^. As shown in Fig. 7a, b, H/R induced the phosphorylation and activation of EGFR, which was further augmented by co-incubation with MMP-10. H/R induced the cleavage of caspase-3 and poly (ADP-ribose) polymerase 1 (PARP-1) in HKC-8 cells, which was prevented by MMP-10 (Fig. 7c–e). However, inhibition of EGFR activation by erlotinib restored the expression of cleaved caspase-3 and PARP-1 (Fig. 7c–e). Similarly, knockdown of HB-EGF by siRNA approach, the EGFR ligand, also restored the expression of cleaved caspase-3 and PARP-1 (Fig. 7f–i). These results suggest that MMP-10-mediated protection of HKC-8 cells against apoptosis is dependent on HB-EGF-mediated EGFR activation.

We also assessed the role of MMP-10-mediated EGFR activation in protecting kidneys after treatment with cisplatin. As shown in Supplemental Fig. S2a–d, the phosphorylation of EGFR (Tyr845), AKT (Ser473), and ERK1/2 (Thr202/Tyr204) was slightly increased in the kidney after cisplatin treatment, which was further enhanced by MMP-10. In cultured kidney epithelial cells (HKC-8), MMP-10 also induced EGFR activation and abolished the induction of cleaved PARP-1 and cleaved caspase-3 in response to cisplatin (Supplemental Fig. S2e–h). Flow cytometry with annexin V-labeling assay also demonstrated that MMP-10 protects tubular cells from apoptosis induced by cisplatin in vitro (Supplemental Fig. S2i and j). Moreover, either inhibition of EGFR activation by erlotinib or knockdown of HB-EGF by siRNA inhibited EGFR activation and restored cleaved caspase-3 and PARP-1 expression (Supplemental Fig. S3). Erlotinib also inhibited PCNA expression (Supplemental Fig. S3a and f). Therefore, EGFR activation plays an important role in mediating MMP-10 protection of tubular cells after injury.

### MMP-10 activates EGFR signaling by proteolytic activation of HB-EGF

**Fig. 8:**
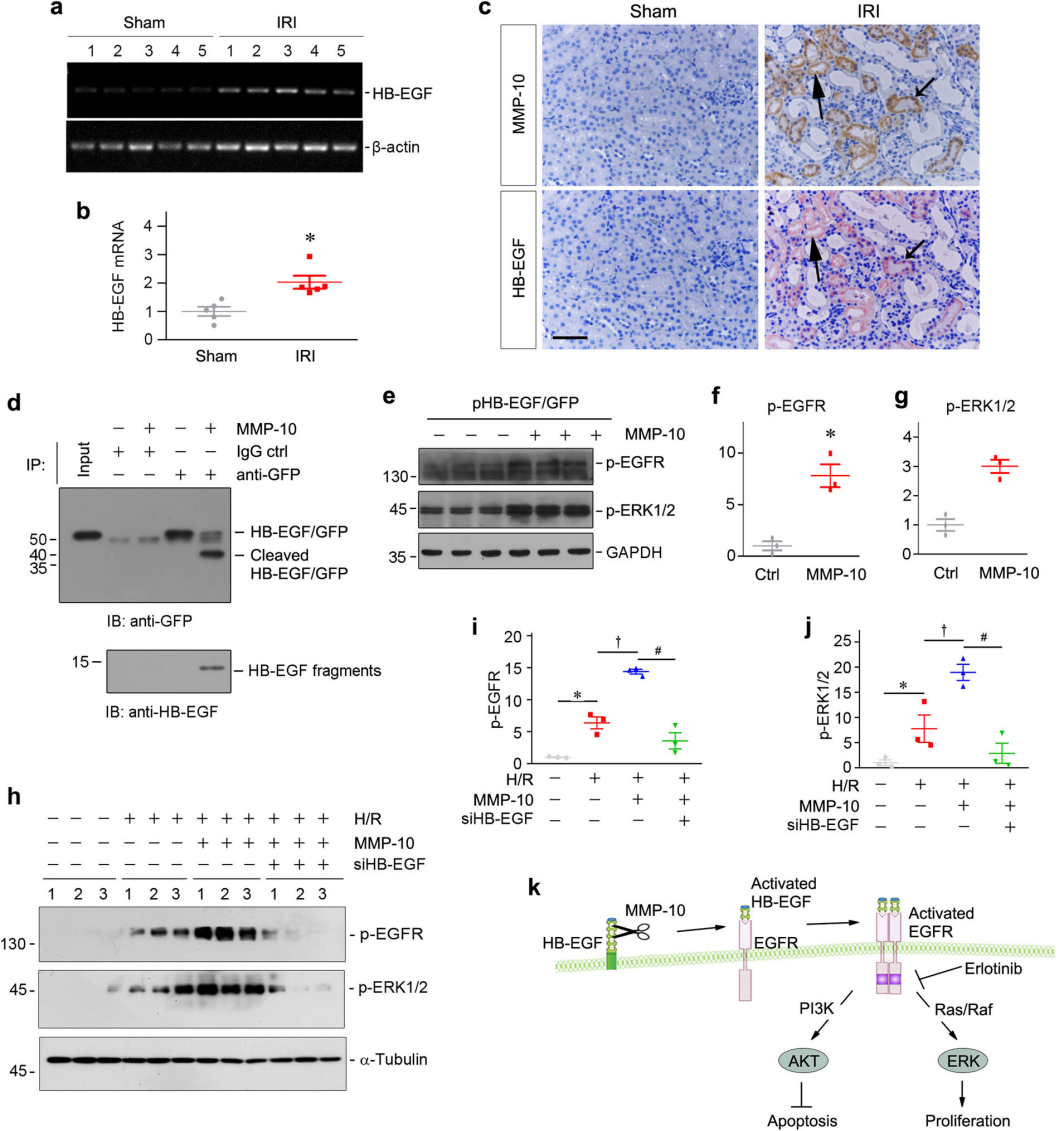
MMP-10 cleaves HB-EGF and activates HB-EGF/EGFR signaling. **a**, **b** RT-PCR results show renal mRNA expression of HB-EGF at 24 h after IRI. Representative RT-PCR results (**a**) and quantitative data (fold induction over the sham control) (**b**) are presented. *P < 0.05 versus sham controls (*n* = 5). **c** Colocalization of MMP-10 and HB-EGF in renal tubules at 24 h after IRI. Kidney serial sections were immunostained for MMP-10 and HB-EGF, respectively. Arrows indicates the colocalization of MMP-10 and HB-EGF in renal tubules. Scale bar, 50 µm. **d** MMP-10 proteolytically cleaves HB-EGF in vitro. HKC-8 cells were transfected with HB-EGF-GFP fusion protein expression vector (pHB-EGF-GFP). The fusion protein in the HKC-8 cell lysates was immunoprecipitated with anti-GFP antibody, followed by incubation with rhMMP-10 for 2 h. After enzymatic cleavage, the supernatant and the beads containing the C-terminal remaining portion of the HB-EGF fusion protein were analyzed by western blot analysis. **e** Representative Western blots show protein expression of p-EGFR (Tyr845) and p-ERK1/2 (Thr202/Tyr204) after MMP-10 treatment in HKC-8 cells. **f**, **g** Graphic presentations show the relative abundances of p-EGFR (**f**) and p-ERK1/2 (**g**) in different groups as indicated. *P < 0.05 versus cells without rhMMP-10 incubation (*n* = 3). **h**–**j** Knockdown of HB-EGF by siRNA abolishes MMP-10-mediated EGFR and ERK1/2 activation. Western blotting (**h**) and quantitative data show protein level of p-EGFR (**i**) and p-ERK1/2 (**j**) after various treatments as indicated. *P < 0.05 versus control cells; ^†^*P* < 0.05 versus H/R alone; ^#^*P* < 0.05 versus H/R with MMP-10 (*n* = 3). **k** Schematic presentation depicts the potential mechanism by which MMP-10 protects against AKI. MMP-10 possesses the ability to cleave HB-EGF, the ligand of EGFR. This leads to the binding of cleaved HB-EGF to EGFR and activates downstream signaling of AKT and ERK, respectively, leading to tubular cell survival and proliferation.

To elucidate how MMP-10 activates EGFR signaling in vivo, we studied HB-EGF, a membrane-anchored form of the EGFR ligands whose activation requires proteolytic cleavage in a process known as ectodomain shedding^30,31^. We first examined the expression of HB-EGF in IRI mice. As shown in Fig. 8a, b, expression of HB-EGF mRNA was induced in the kidneys after IRI. Furthermore, consistent with in vitro data (Fig. 7f, g), renal HB-EGF protein was also upregulated after IRI and predominantly localized at the survived tubules (Fig. 8c). Immunostaining for both MMP-10 and HB-EGF on the serial sections confirmed their colocalization in renal tubules after IRI (Fig. 8c), suggesting their intrinsic connection.

To ascertain MMP-10 activation of HB-EGF via ectodomain shedding, we tested whether MMP-10 can proteolytically cleave HB-EGF. To this end, we transfected HKC-8 cells with GFP-tagged HB-EGF expression vector (pHB-EGF-GFP), followed by incubation with MMP-10. Cell lysates were then immunoprecipitated with anti-GFP antibody. As shown in Fig. 8d, MMP-10 clearly cleaved the HB-EGF-GFP fusion protein (52 kDa) to produce a truncated form with 40 kDa in size. Furthermore, a soluble fragment (~12 kDa) of HB-EGF was readily detected in the supernatant by western blot analyses (Fig. 8d), suggesting that MMP-10 cleaves HB-EGF and releases soluble HB-EGF fragments.

Finally, we tested whether the soluble HB-EGF fragments released by MMP-10 is biologically active and activates EGFR signaling. To this end, HKC-8 cells transfected with HB-EGF-GFP expression vector were incubated with MMP-10. As shown in Fig. 8e–g, MMP-10 promoted activation of EGFR and ERK1/2 in these cells. Furthermore, knockdown of endogenous HB-EGF by siRNA approach abolished EGFR and ERK1/2 activation induced by hypoxia/reoxygenation and MMP-10 treatment (Fig. 8h–j). Taken together, these data suggest that MMP-10 cleaves HB-EGF to produce active fragments, leading to activation of EGFR signaling (Fig. 8k).

## Discussion

In this study, we have provided evidence showing that MMP-10 is induced in renal tubular epithelium after AKI and it functionally protects against kidney damage triggered by ischemic and nephrotoxic insults. Mechanistically, MMP-10 possesses the ability to proteolytically cleave HB-EGF, the ligand of EGFR (Fig. 8). As summarized in Fig. 8, this leads to the liberation and release of HB-EGF and triggers its binding to EGFR and activates its downstream signaling of AKT and ERK1/2. Such a cascade of events finally leads to the protection of tubular cells from apoptosis following injury and promotion of tubular repair and regeneration by augmenting cell proliferation (Fig. 8). Therefore, our studies for the first time identify HB-EGF as a previously unrecognized substrate of MMP-10 and provide a novel connection between MMP-10 and EGFR signaling. These findings also offer unique and significant insights into the mechanism by which MMP-10 protects kidneys against AKI in the preclinical setting.

In many other tissues, such as the liver and muscles, earlier studies have shown that MMP-10 is induced after injury and it promotes tissue repair and regeneration^32–34^. Several studies in the oncology field also support a role for increased MMP-10 in promoting epithelial growth^35,36^. In this regard, the role of MMP-10 in promoting tubular repair and regeneration after AKI is not completely surprising, and it probably represent a common protective response of different organs following injury. Consistent with this view, a recent study also observes the upregulation of MMP-10 in mouse kidney after IRI using a microarray assay^37^, although the function of MMP-10 was not investigated. We show herein that MMP-10 not only promotes tubular repair by inducing cell proliferation but also protects tubular cells from apoptosis after either ischemic or nephrotoxic insults both in vitro and in vivo, highlighting its prevention of tubular injury and death in the first place. The combined effects of MMP-10 on tubular cell survival and proliferation render it a potent factor that dictates the outcome of the injured kidneys.

It should be stressed that these same effects of MMP-10 on cell survival and proliferation may lead to disparate outcomes in different settings. For instance, the increased expression of MMP-10 is associated with poor prognosis and decreased survival of patients with RCC^17^. In that regard, MMP-10 is detrimental by promoting renal cancer cell invasion and progression. Although the underlying mechanism by which MMP-10 promotes RCC progression was not investigated in that study, the potential activation of EGFR and its downstream signaling as identified in the present study could well explain for its disadvantageous action of MMP-10 in RCC.

The most interesting and novel finding of the present study is the identification of HB-EGF as new substrate of MMP-10 in AKI setting. As a unique EGFR ligand, HB-EGF is synthesized as transmembrane protein, and its activation depends upon the proteolytical cleavage of its extracellular domain, a process known as the ectodomain shedding. Previous studies suggest that HB-EGF ectodomain cleavage is mediated by the ADAM (a disintegrin and metalloproteinase) family of endopeptidases to release soluble mature peptide, which then acts in a paracrine or autocrine fashion^38^. However, our results herein indicate that MMP-10 plays an essential role in mediating the ectodomain shedding of HB-EGF and its activation in the setting of AKI. This conclusion is supported by several lines of evidence. First, in vivo evidence suggests that both MMP-10 and HB-EGF are induced and colocalized in renal tubular epithelium after AKI. In addition, in vitro studies demonstrate that MMP-10 can cleave HB-EGF and release active fragments, leading to the activation of EGFR signaling. Furthermore, either inhibition of EGFR by erlotinib or knockdown of endogenous HB-EGF abolishes MMP-10-mediated protection of tubular epithelial cells (Fig. 7 and Supplemental Fig. S3). These findings clearly point to a key role for MMP-10 in mobilizing and activating HB-EGF, a growth factor that is well known for its involvement in promoting renal epithelial cell repair, proliferation and regeneration after AKI^28,39,40^.

The cleavage of HB-EGF by MMP-10 suggests that this endopeptidase participates in regulating kidney injury and recovery after AKI by activating a potent EGFR ligand, highlighting a unique role for MMP-10 in converting a transmembrane growth factor into its soluble form. Such a cleaved HB-EGF then becomes bioactive and triggers EGFR phosphorylation and its downstream AKT and ERK1/2 activation. EGFR signaling is well known to play a critical role in promoting cell survival and proliferation during tissue repair and regeneration, and depletion of EGFR in renal proximal tubular epithelial cells delay recovery from AKI^22^. Consistently, inhibition of EGFR signaling by erlotinib, a small molecule inhibitor of EGFR tyrosine kinase^41^, abolishes renal protection in vivo and in vitro elicited by MMP-10 (Fig. 6, Fig. 7 and Supplemental Fig. S3). It should be pointed out that a single dose of erlotinib is used in the present study in vivo and this finding needs to be substantiated with multiple doses of erlotinib in the future. Furthermore, EGFR binds to several ligands including transforming growth factor-α and amphiregulin^42,43^, which also depend on ectodomain shedding for activation. Whether MMP-10 also plays a role in mediating the activation of other EGFR ligands remains to be investigated.

In summary, we show herein that MMP-10 is an important regulator of renal structure and function after AKI. MMP-10 protects kidneys from injury by inhibiting tubular cell apoptosis and promoting cell proliferation via activating EGFR signaling. Our results identify HB-EGF as a novel substrate of MMP-10. As such, MMP-10 promotes the ectodomain shedding of HB-EGF, leading to its liberation and release, which then binds to EGFR and triggers EGFR-mediated repair and regenerative program. Therefore, modulation of MMP-10/HB-EGF/EGFR signaling axis may offer novel strategies in designing therapeutics against AKI.

## Supplementary information

Figure S1

Figure S2

Figure S3

Supplementary Figure legend

Supplementary Tables
