# Supplementary figures and images for: Matrix metalloproteinase-10 protects against acute kidney injury by augmenting epidermal growth factor receptor signaling
Source: Cell Death Dis. 2021 Jan 12;12(1):70. doi: 10.1038/s41419-020-03301-3 (PMC7803968; doi:10.1038/s41419-020-03301-3)

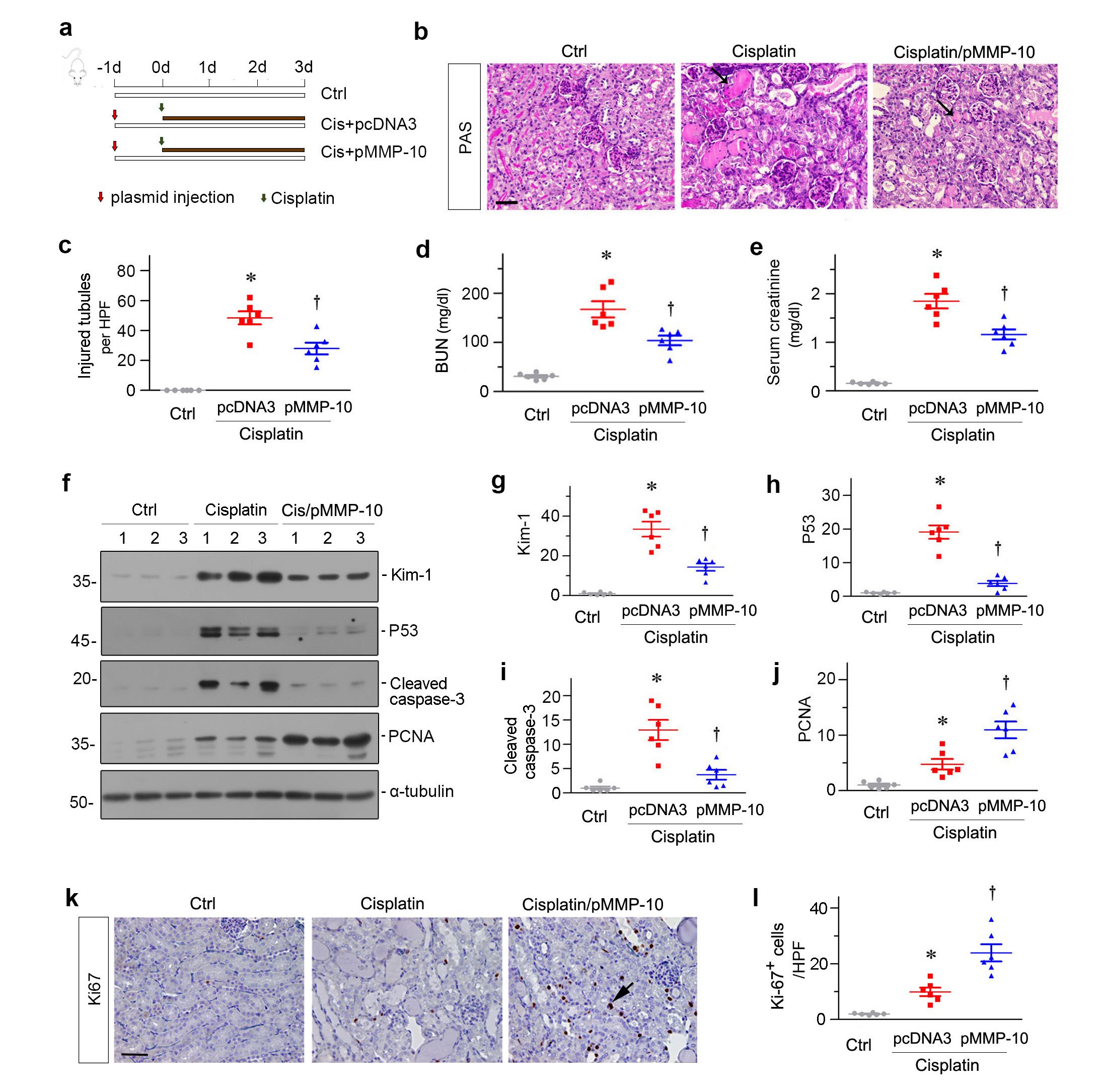

Supplement: Supplementary file 1 — Figure S1 [file 41419_2020_3301_MOESM1_ESM.jpg]

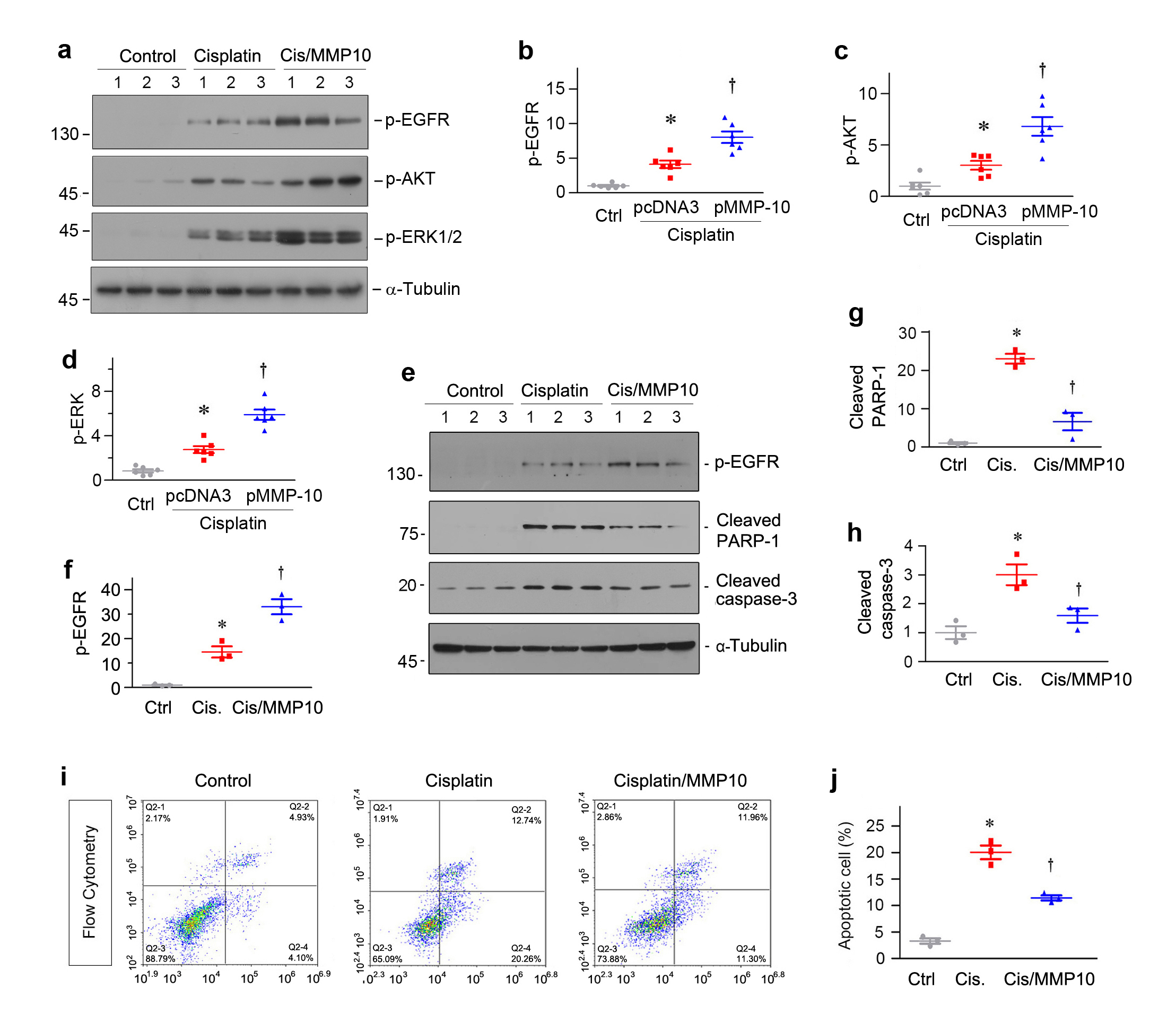

Supplement: Supplementary file 2 — Figure S2 [file 41419_2020_3301_MOESM2_ESM.jpg]

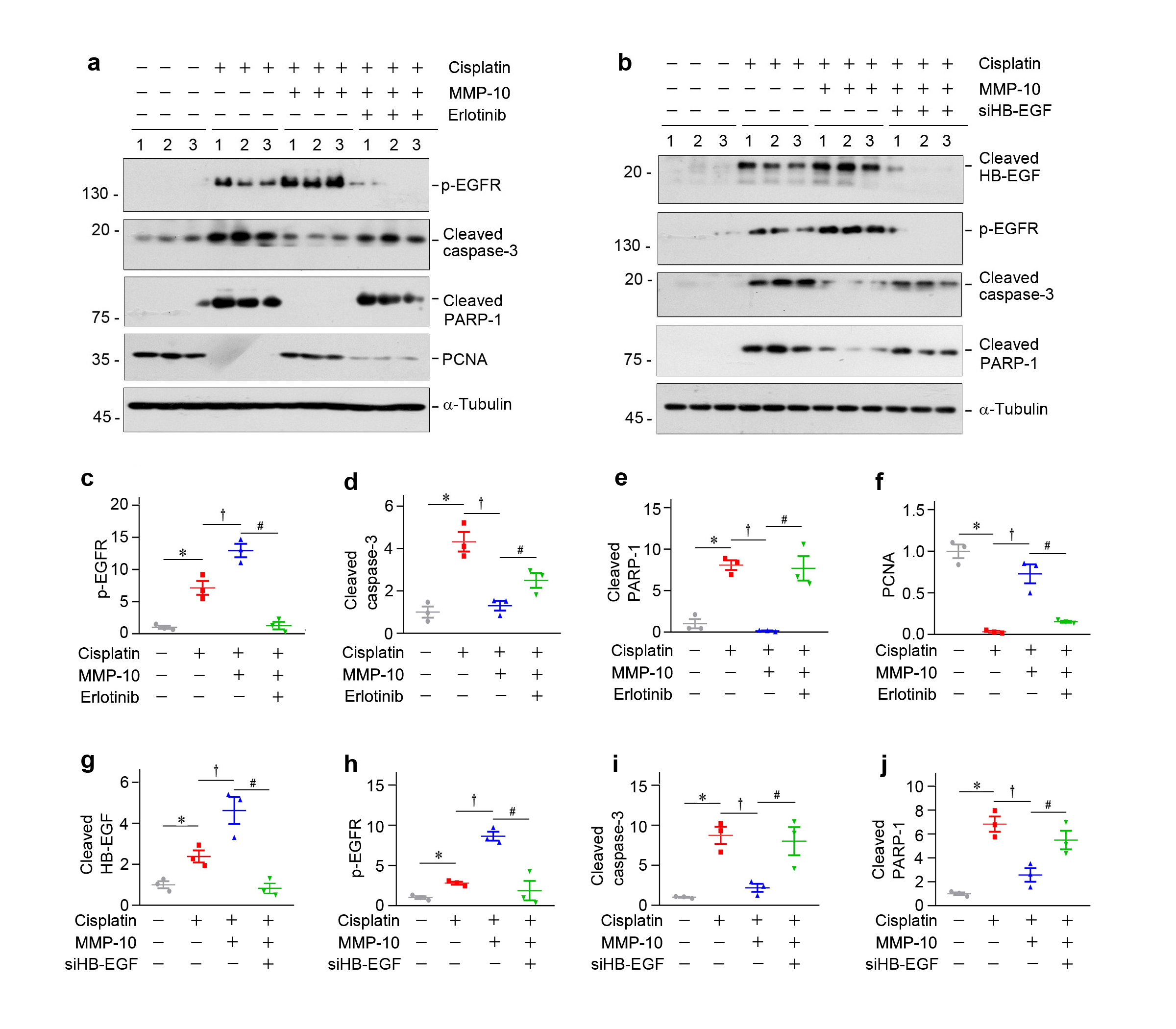

Supplement: Supplementary file 3 — Figure S3 [file 41419_2020_3301_MOESM3_ESM.jpg]
