# Supplementary material for: Matrix metalloproteinase-10 protects against acute kidney injury by augmenting epidermal growth factor receptor signaling
Source: Cell Death Dis. 2021 Jan 12;12(1):70. doi: 10.1038/s41419-020-03301-3 (PMC7803968; doi:10.1038/s41419-020-03301-3)
Supplement: Supplementary file 4 — Supplementary Figure legend [file 41419_2020_3301_MOESM4_ESM.doc]

**Supplementary Figure legend**

**Fig. S1. Exogenous MMP-10 protects against nephrotoxic AKI induced by cisplatin.** (**a**) Experimental design. Red arrows indicate the injection of pcDNA3 or pFlag-MMP-10 plasmid. Green arrows indicate the timing of cisplatin injection. (**b**) Representative micrographs show kidney morphology in different groups as indicated. Kidney sections were subjected to PAS staining. Arrows indicate injured tubules. Scale bar, 50 µm. (**c**) Quantitative determination of injured tubules in different groups as indicated. At least 10 randomly selected fields were evaluated and results were averaged for each kidney. **P* < 0.05 versus controls (n=6); †*P* < 0.05 versus cisplatin (n=6). (**d**, **e**) Graphic presentations show blood urea nitrogen (BUN) (**d**) and serum creatinine (**e**) levels in different groups as indicated. **P* < 0.05 versus control subjects (n=6); †*P* < 0.05 versus cisplatin (n=6). (**f**) Representative Western blot analyses show renal expression of Kim-1, p53, cleaved caspase 3 and PCNA proteins in different groups as indicated. Numbers (1, 2, and 3) indicate each individual animal in a given group. (**g**-**j**) Graphic presentations show the relative levels of Kim-1 (**g**), p53 (**h**), cleaved caspase 3 (**i**) and PCNA (**j**) expression in different groups as indicated. **P* < 0.05 versus control subjects (n=6); †*P* < 0.05 versus cisplatin (n=6). (**k**, **l**) MMP-10 promotes cell proliferation in the kidney after cisplatin injection. Representative micrographs (**k**) and quantitative data (**l**) show Ki-67-positive cells in different groups as indicated. **P* < 0.05 versus control subjects (n=6); †*P* < 0.05 versus cisplatin (n=6).

**Fig. S2. MMP-10 protects against tubular cell apoptosis induced by cisplatin. (a)** Representative Western blots show protein expression of p-EGFR (Tyr845), p-AKT (Ser473) and p-ERK1/2 (Thr202/Tyr204) in the kidney after various treatments as indicated. (**b-d**) Graphic presentations show the relative abundances of p-EGFR (**b**), p-AKT (**c**) and ERK1/2 (**d**) in different groups as indicated. ^*^*P* < 0.05 versus controls; ^†^*P* < 0.05 versus cisplatin alone (n=6). (**e**) Representative Western blots show protein expression of p-EGFR, cleaved PARP-1 and cleaved caspase 3 after various treatments in HKC-8 cells. (**f-h**) Graphic presentations show the relative abundances of p-EGFR (**f**), cleaved PARP-1 (**g**) and cleaved caspase 3 (**h**) proteins in different groups as indicated. ^*^*P* < 0.05 versus control cells; ^†^*P* < 0.05 versus cisplatin alone (n=3). (**i**) Representative flow cytometry analyses show that MMP-10 reduced cisplatin-induced cell apoptosis. HKC-8 cells were pre-incubated with recombinant human MMP-10 protein (100 ng/ml) for 1 hour, followed by incubation with cisplatin (25 µg/ml) for 24 hours. (**j**) Graphic presentation shows the percentage of apoptotic cells in different groups as indicated. HKC-8 cells were treated with cisplatin in the absence or presence of MMP-10. The phycoerythrin-labeled annexin V (PE-A)-positive cells were counted by flow cytometry. ^*^*P* < 0.05 versus control cells; ^†^*P* < 0.05 versus cisplatin alone (n=3).

**Fig. S3. Blockade of EGFR activation by erlotinib or knockdown of HB-EGF restores tubular cell apoptosis in response to cisplatin.** (**a**, **b**) Representative Western blots show that EGFR inhibitor erlotinib (**a**) or knockdown of HB-EGF (**b**) inhibited EGFR activation and abolished MMP-10-mediated protection of HKC-8 cells after cisplatin treatment. Western blots show the expression of cleaved PARP-1 and caspase 3 in HKC cells in response to cisplatin. (c-j) Graphic presentations show the relative abundances of p-EGFR (**c**, **h**), cleaved caspase 3 (**d**, **i**), cleaved PARP-1 (**e**, **j**), PCNA (**f**), and cleaved HB-EGF (**g**). ^*^*P* < 0.05 versus control cells; ^†^*P* < 0.05 versus cisplatin alone; ^#^*P* < 0.05 versus cisplatin plus MMP-10 (n=3).
