# Supplementary material for: Matrix metalloproteinase-10 protects against acute kidney injury by augmenting epidermal growth factor receptor signaling
Source: Cell Death Dis. 2021 Jan 12;12(1):70. doi: 10.1038/s41419-020-03301-3 (PMC7803968; doi:10.1038/s41419-020-03301-3)
Supplement: Supplementary file 5 — Supplementary Tables [file 41419_2020_3301_MOESM5_ESM.doc]

| Antibodies | Catalogue number | Company | Location |
| --- | --- | --- | --- |
| Goat polyclonal anti-MMP-10 (for IHC) | AF-910 | R&D SYSTEMS | Minneapolis, MN |
| Mouse monoclonal anti-MMP-10 (for WB) | sc-80197 | Santa Cruz Biotechnology | Santa Cruz, CA |
| Rabbit polyclonal anti-Kim-1 | BA3537 | Boster Biological Technology, | Wuhan, China |
| Mouse monoclonal anti-PCNA | sc-56 | Santa Cruz Biotechnology | Santa Cruz, CA |
| Rabbit monoclonal anti-Ki-67 | ab16667 | Abcam | Cambridge, MA |
| Mouse monoclonal anti-HB-EGF | ab66792 | Abcam | Cambridge, MA |
| Rabbit polyclonal anti-p-EGFR (Tyr845)  Rabbit monoclonal anti-EGFR | 2231S  ab52894 | Cell Signaling Technology  Abcam | Cambridge, MA  Cambridge, MA |
| Rabbit monoclonal anti-p-ERK1/2 (Thr202/Tyr204)  Rabbit monoclonal anti-ERK1/2 | 9101S  4695S | Cell Signaling Technology  Cell Signaling Technology | Danvers, MA  Danvers, MA |
| Rabbit monoclonal anti- p-AKT (Ser473）  Rabbit polyclonal anti-AKT | 4060S  9272S | Cell Signaling Technology  Cell Signaling Technology | Danvers, MA  Danvers, MA |
| Rabbit polyclonal anti-caspase 3 | 9662S | Cell Signaling Technology | Danvers, MA |
| Rabbit polyclonal anti-PARP-1 | 9542S | Cell Signaling Technology | Danvers, MA |
| Mouse monoclonal anti-P53 | sc-126 | Santa Cruz Biotechnology | Santa Cruz, CA |
| Mouse monoclonal anti-mature HB-EGF  Mouse monoclonal anti-GAPDH | sc-74526  RM2002 | Santa Cruz Biotechnology  Ray Antibody Biotech | Santa Cruz, CA  Beijing, China |
| Mouse monoclonal anti-β-actin | RM2001 | Ray Antibody Biotech | Beijing, China |
| Mouse monoclonal anti-α-tubulin | RM2007 | Ray Antibody Biotech | Beijing, China |

**Supplementary Table S1. The sources of antibodies used in this study**

**Supplementary Table S2. Nucleotide sequences of the primers used for PCR**

| Mouse | Primer Sequence 5’to 3’ | | | |
| --- | --- | --- | --- | --- |
| gene | Forward | | Reverse | |
| MMP-10 | | GACCCCACTCACTTTCTCCA | | GGAATAAGTTGGTCCCTGAGG |
| HB-EGF | | AGACCCATGCCTCAGGAAAT | | ACGACAGTACTACAGCCACC |
| β-actin | | GAGCGCAAGTACTCTGTGTG | | AACGCAGCTCAGTAACAGTC |
